# Geographical Distribution of Osteopathic Neurosurgery Residents

**DOI:** 10.7759/cureus.68491

**Published:** 2024-09-02

**Authors:** Ogechukwu Ariwodo, Andrew Greek, Ryan Wong, Vincent S Alexander, Andrew D Vogel, Bracken Burns, Kristen Conrad-Schnetz

**Affiliations:** 1 Department of Neurosurgery, Philadelphia College of Osteopathic Medicine South Georgia, Moultrie, USA; 2 Department of Medicine, Kansas City University College of Osteopathic Medicine, Joplin, USA; 3 Department of Medicine, Nova Southeastern University Dr. Kiran C. Patel College of Osteopathic Medicine, Fort Lauderdale, USA; 4 Department of Research, Alabama College of Osteopathic Medicine, Dothan, USA; 5 Department of Surgery, East Tennessee State University, Johnson City, USA; 6 Department of Surgery, Cleveland Clinic South Pointe Hospital, Cleveland, USA

**Keywords:** region, residency, match, state, neurosurgery, osteopathic

## Abstract

Introduction: The neurosurgery residency match has grown increasingly competitive, especially for osteopathic (DO) medical students, amidst the transition to a single accreditation system in 2020. This shift required former American Osteopathic Association (AOA) programs to apply for Accreditation Council for Graduate Medical Education (ACGME) accreditation, leading to a notable reduction in programs with a history of accepting DO applicants. This study aims to explore both potential geographical trends in residency match among recent DO neurosurgical residents and in the number of DO neurosurgical residents pre- and post-ACGME merger.

Methods: Neurosurgery residency programs during the 2023-2024 academic year were identified, and each program's residents, resident degrees, and resident post-graduate years were collected from residency programs' websites. Descriptive statistics were used to analyze the ratios of DO and allopathic (MD) residents, while regression analyses were used to determine the trends in DO residents between 2017 and 2024. DO residents were also collated by state to observe their geographical distribution.

Results: A comprehensive cross-sectional analysis of 115 neurosurgery residency programs across the United States from 2016 to 2024 reveals a significant decrease in DO residents, from 14 in 2016 to four in 2024, with an average of six DO residents per year post-merger. A geographical heatmap analysis pinpointed New Jersey, Michigan, and California as states with the highest proportions and numbers of DO neurosurgery residents.

Conclusion: These findings show the geographical distribution of DO neurosurgery residents in the US. Recognizing and understanding these geographical trends could be essential in the strategic application planning for DO candidates and the need for residency programs to reassess selection criteria to be more inclusive of DO applicants.

## Introduction

Neurosurgery has historically been a competitive specialty to match for both allopathic (MD) and osteopathic (DO) medical students. Before 2020, DO applicants could match through the American Osteopathic Association (AOA) or the National Resident Matching Program (NRMP). The AOA match facilitated residency placement into traditional DO programs. As of 2020, the residency match moved to a single accreditation system. Former AOA programs were required to apply to the Accreditation Council for Graduate Medical Education (ACGME) to continue training residents. As a result, many former AOA surgical residencies have closed, leading to fewer residency programs with a strong history of accepting DO applicants [1]. Only four of the 10 former AOA neurosurgical programs transitioned to ACGME accreditation [2]. With the recent establishment of a unified graduate medical education system in 2020 and an increase in DO schools, concerns arose regarding how this would impact the match rates for DO students in surgical subspecialties [3,4].

As competition for residency positions increases, factors such as geographical residency placement trends become increasingly critical and warrant consideration [5]. It has been consistently demonstrated in other surgical subspecialties that a significantly higher proportion of applicants match at programs affiliated with or located in the same region as their medical school [6-8]. A solid understanding of locations that historically accept DO students into a neurosurgery residency may prove beneficial when applying. However, there has been no research on geographical placement trends in the neurosurgery residency match for osteopathic students. With few DO schools having an affiliated neurosurgical program, comprehension of these patterns will provide valuable insights for future DO neurosurgical applicants [9].

This study aims to explore potential geographical residency match trends among recent DO neurosurgical residents while also examining the trend in the number of DO neurosurgical residents over the past seven years. This period encapsulates data before and after the AOA/ACGME merger, allowing insight into how the merger affected these trends. Hopefully, this exploration will contribute to the ongoing discourse surrounding the evolving landscape of the neurosurgical match.

## Materials and methods

All accredited neurosurgery residency programs in the United States were identified for the 2023-2024 academic year in November 2023. Residents, their post-graduate year (PGY), degrees, and the program's state were collected from each program's website. Websites with up-to-date resident information were included for analysis. Up-to-date information was confirmed by either explicit indication of the current academic year or cross-referenced with publicly available resident data online. This included reporting the graduation year or a professional profile via LinkedIn, Doximity, or X (formerly known as Twitter). Data from the 2024 match year were obtained from publicly available NRMP data [10]. Residents were differentiated by degree. DO residents were then further stratified by their post-graduate years to observe the trends in the number of neurosurgery residents over the past seven years. The DO match trends in neurosurgery and geographical distribution within neurosurgery residency programs across the United States were analyzed. A geographical heatmap for the density of DO neurosurgery residents by state was generated using Google Sheets. Descriptive statistics and regression analysis were performed using GraphPad Prism 10 (GraphPad Software, San Diego, California). A p-value of < 0.05 was considered significant.

## Results

A total of 115 up-to-date neurosurgery residency program websites were identified. Of these programs, there were 1,623 residents in the 2023-2024 academic year. The majority (n = 1,558, 96%) had an MD degree. Nine residents (0.55%) had a Bachelor of Medicine and Bachelor of Surgery (MBBS) degree, and 56 residents (3.45%) were found to hold a DO degree from a total of 18 neurosurgery programs (15.65%).

**Figure 1:**
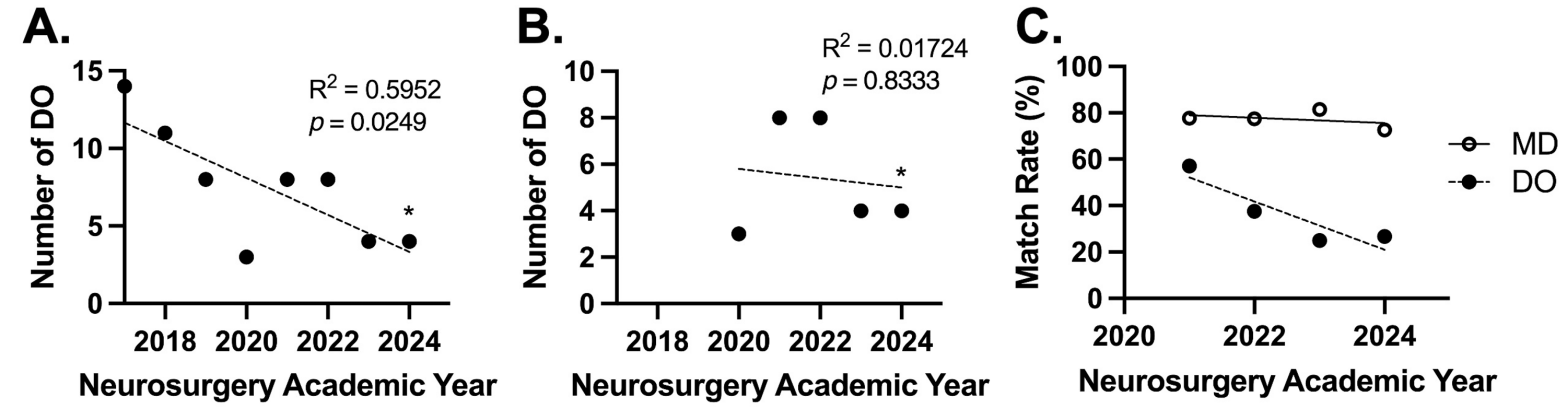
Trends in the number of PGY-1 osteopathic (DO) neurosurgery residents over time (A) Total number of neurosurgery residents per year. (B) Trends in the number of neurosurgery residents per year, post-American Osteopathic Association/Accreditation Council for Graduate Medical Education residency merge. (C) Comparison in neurosurgery match rates for osteopathic (DO) and allopathic (MD) applicants between 2021 and 2024. *Denotes data observed from the 2024 National Residency Matching Program match results [10].

The number of PGY-1 DO neurosurgery residents has significantly decreased from 14 in 2016 to four in 2024 (R^2^ = 0.5382, p = 0.0249) (Figure 1A). The number of DO neurosurgery residents was found to remain constant between 2020 and 2024 (R^2^ = 0.01724, p = 0.8333), with an average of six residents per year (Figure 1B). There have been fewer than 10 new DO neurosurgery residents each match year since the AOA/ACGME merger. Additionally, the match rate for DO applicants decreased from 57.14% to 26.67%, while that of MD applicants has remained steady between 72.57% and 81.5% (Figure 1C).

**Figure 2:**
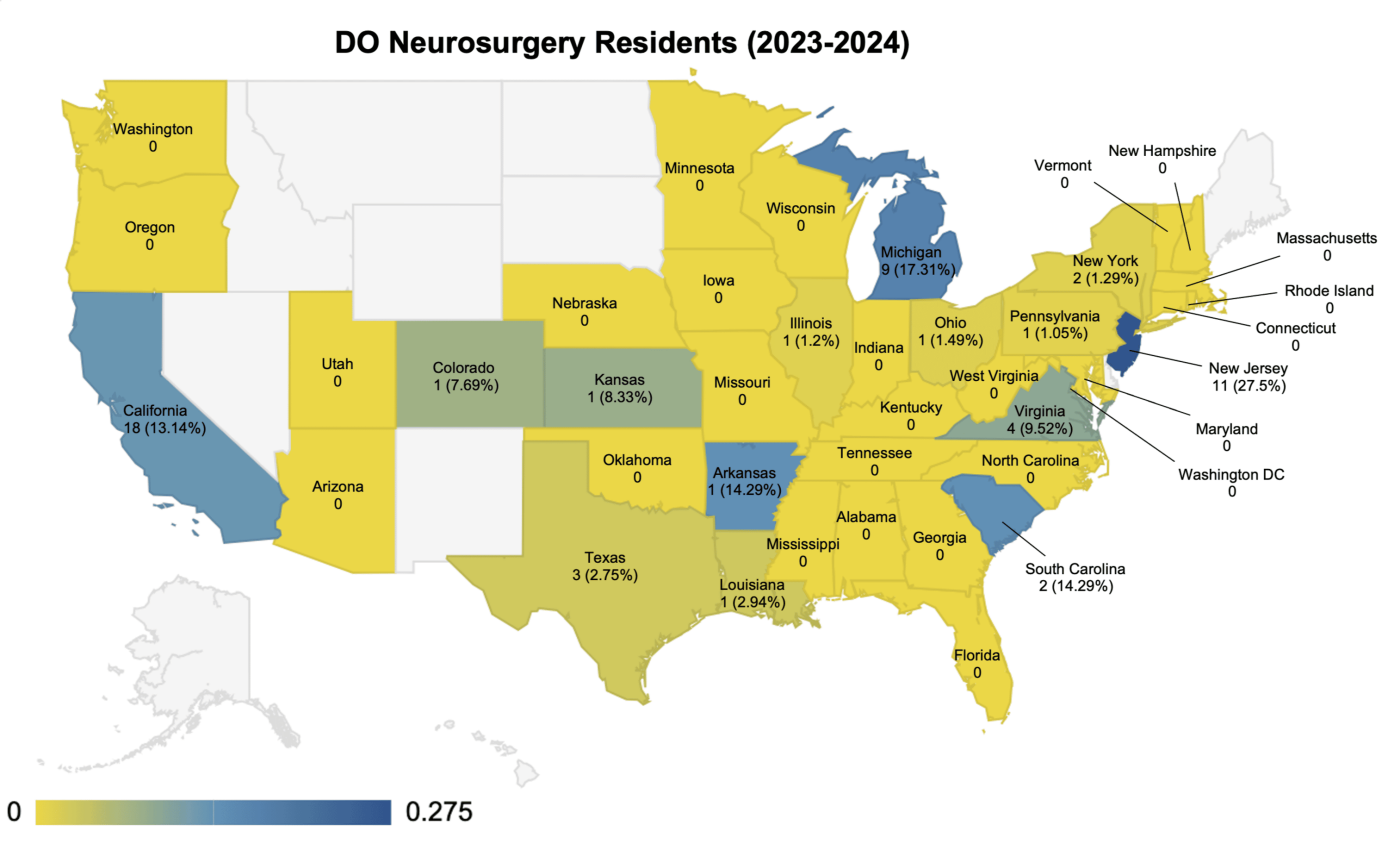
Geographical distribution of osteopathic (DO) neurosurgery residents for the 2023-2024 academic year White indicates no data. Dark blue corresponds to 27.5%; yellow corresponds to 0%. Values are labeled as n (%).

Figure 2 shows the geographical distribution of DO neurosurgery residents. New Jersey had the highest proportion of DO neurosurgery residents (n = 11, 27.5%); Michigan (n = 9, 17.3%) had the second highest; and Arkansas (n = 1, 14.29%) and South Carolina (n = 2, 14.29%) were the third highest. When looking at the total number of DO neurosurgery residents by state, California had the highest number (n = 18, 13.14%); New Jersey had the second highest (n = 11); and Michigan had the third highest (n = 9). In total, 26 states (67%) with neurosurgery residency programs had no DO neurosurgery residents. Several states with no neurosurgery residency programs were excluded from the analysis, including Alaska, Hawaii, Idaho, Nevada, Montana, Wyoming, North Dakota, South Dakota, Delaware, and Maine.

## Discussion

This study has explored the geographical distribution and trends of DO neurosurgery residents in the United States, revealing significant insights into the shifting landscape of neurosurgery residency placements. Brazdzionis et al. reported that although there has been a match rate increase for all DO candidates post-AOA/ACGME merger, this trend does not correspond to certain surgical subspecialties [2]. The results from this study showed that the decrease in the number of incoming DO neurosurgery residents from 2016 to 2024, along with the consistent numbers of total DO neurosurgery residents post-AOA/ACGME merger, further supported the findings of Brazdzionis. Additionally, the match rate for DO neurosurgery applicants has been decreasing from 57.14% to 26.67%, while that of MD applicants has remained steady between 72.57% and 81.5%. Prior to the single accreditation, there were 11 AOA-accredited neurosurgery residency programs with 126 positions available to DO neurosurgical candidates [11]. Eight of the 11 programs applied for ACGME accreditation, and only four were accredited [4,12]. The geographical location of the four programs that received accreditation matched the geographical distribution of DO residents in Figure 2 [12]. Notably, the geographical heatmap illustrates a concentrated distribution of DO residents in certain states, showing an overall variance in state-by-state distribution, with California constituting the highest number of DO neurosurgery residents while New Jersey and Michigan had the highest percentage of DO neurosurgery residents. This observation might reflect some factors influencing match outcomes that have already been documented in the literature, such as the regional or institutional preferences of DO candidates to formerly AOA-accredited programs, affiliations, and the geographical proximity of DO medical schools to residency programs [13-16].

At the time of data analysis, over 80% of neurosurgery residency programs did not have a DO resident, and 26 states (67%) with neurosurgery residency programs had no DO neurosurgery residents. This finding is in line with studies conducted by Beckman et al. and Panchal et al. that reported many surgical subspecialty residency programs, including neurosurgery, having a low percentage of DO residents, and some programs even disqualifying DO applicants from training at their institutions [17,18]. Factors and biases such as these emphasize the importance of understanding geographical distribution trends, as DO students might be more inclined to apply to programs in states or regions with current DO residents or a record of graduating DO residents. These programs could be perceived as more welcoming to osteopathic candidates, potentially affecting the nationwide distribution of DO neurosurgery residents.

The transition of formerly AOA programs to a single accreditation system may have also influenced the results of our analysis. There was a notable significant decline in the number of PGY-1 DO neurosurgery residents per year over the past eight years. The loss of seven neurosurgical programs, as found by Cummings et al., highlights the tangible impact of a single accreditation system on the availability of residency positions specifically tailored for DO graduates [4]. This reduction in specialized training opportunities necessitates reevaluating how DO neurosurgery applicants navigate the matching process and the geographical implications of these systemic changes.

Furthermore, Panchal et al. conducted a study that showed most program directors of ACGME programs that were never AOA-accredited recommended DO students to take both United States Medical Licensing Examination (USMLE) Step 1 and Step 2 Clinical Knowledge (CK), especially for competitive specialties such as neurosurgery [18]. This, in turn, places a significant financial and logistical burden on these applicants. This might influence DO students' geographical choices, pushing them toward programs with a history of AOA accreditation or those known to value the Comprehensive Osteopathic Medical Licensing Examination (COMLEX-USA) scores equally.

The financial implications of multiple licensing exams and the importance of audition rotations, particularly in formerly AOA-accredited programs, could further complicate the geographical spread of DO neurosurgery applicants, potentially reinforcing regional biases in the matching process.

Addressing the potential regional biases shown in this study aligns with addressing match success rates. There is a noted underrepresentation of DOs in leadership roles (residency program director) within specific surgical subspecialties such as neurosurgery. Brazdzionis et al. noted that this underrepresentation significantly correlated with lower match rates for DO students in these specialties [2]. Bello et al. conducted a study that showed the presence of DO faculty as the only factor that was independently associated with the integration of DO residents at 39 general surgery residencies without a previous DO resident [19]. Studies have shown that factors such as robust research programs, strong mentorship, specialty interest groups, and early exposure to subspecialties make candidates applying to these residencies very competitive and significantly increase a candidate's match prospects [20,21]. Enhancing research involvement at osteopathic medical schools via in-person or remote opportunities, particularly in surgical subspecialties, could improve DO candidates' success in neurosurgery. Gupta et al. showed an increase in the number of publications neurosurgery residency applicants have before applying. This trend could put applicants from less research-focused institutions at a disadvantage. Additionally, it may convince more students to take a research year to improve their match odds, disadvantaging those without the financial means to do so [22].

Mentorship is crucial for medical students aiming for successful residency matches, particularly in neurosurgery. Mentors provide essential guidance, support, and insights, significantly enhancing students' match prospects. A solid mentor-mentee relationship has been linked to higher match rates, highlighting its importance in medical education and the need for surgical organizations to facilitate mentor-student connections [21,23]. Advocacy for an equitable recognition of DO training and board scores, along with promoting early exposure to surgical subspecialties through medical school interest groups and faculty, could also aid in improving DO students' competitiveness in neurosurgery residency applications.

Limitations

Our study's limitations include a reliance on publicly available data from residency program websites, which, while valuable, may not always be accurate or exhaustive. Additionally, the post-AOA/ACGME merger into a single accreditation system is still relatively new and, as a result, could have influenced the trends observed, warranting further investigation into the long-term effects of the AOA/ACGME merger on DO neurosurgery placements.

## Conclusions

This study provides critical insights into the geographical distribution and evolving trends of DO neurosurgery residents in the United States, which are vital for both program directors and applicants when planning their applications. Future research should explore the underlying causes of these trends, including program preferences, potential biases, and the broader implications of the unified graduate medical education system on DO applicants in surgical specialties.
